# Are medical students confident in taking a sexual history? An assessment on attitude and skills from an upper middle income country

**DOI:** 10.1186/s13104-015-1220-y

**Published:** 2015-06-17

**Authors:** Farnaza Ariffin, Ken Lee Chin, ChirkJenn Ng, Maizatullifah Miskan, Verna KarMun Lee, Mohammad Rodi Isa

**Affiliations:** Department of Family Medicine, Faculty of Medicine, Universiti Teknologi MARA, Sungai Buloh, Malaysia; Department of Pharmacy Practice, Faculty of Pharmacy, Universiti Teknologi MARA, Shah Alam, Malaysia; Department of Primary Care Medicine, Faculty of Medicine, University of Malaya, Kuala Lumpur, Malaysia; Department of Family Medicine, Faculty of Medicine, International Medical University, Bukit Jalil, Malaysia; Department of Community Medicine, Faculty of Medicine, Universiti Teknologi MARA, Shah Alam, Malaysia

**Keywords:** Sexual history, Medical students, Undergraduate, Attitude, Perception, Skills, Training

## Abstract

**Background:**

Sexual history training during undergraduate education is essential for preparing future doctors to handle patients’ sexual health concerns. The purpose of this study was to assess the attitudes and perceptions of final-year medical students in Malaysia toward sexual history taking and the training they receive from their medical schools.

**Methods:**

The study used a cross-sectional survey of 379 final-year medical students from three medical schools in Malaysia. Students were asked to rate their attitudes and perceptions regarding training on taking sexual histories using a newly developed questionnaire with good internal consistency (Cronbach’s alpha = 0.73). Ethics approval was obtained from the relevant medical schools, and the statistical analysis was conducted using SPSS, Version 20.0.

**Results:**

The mean age of participants was 23.58 ± 0.65 SD. Participants reported high interest in sexual health and felt it was important for doctors to know how to take a sexual history (95%). Among the participants, only half felt comfortable in taking sexual histories from patients. The participants identified cultural and religious differences between the doctor and the patient as a potential barrier for discussing sexual health. Participants were aware of their own practice and ability, as well as their limitations, in taking sexual histories. Less than half (46%) felt that the training they received adequately prepared them to take sexual histories.

**Conclusions:**

This study identified gaps in sexual health training among medical schools in Malaysia. The delivery of sexual health education program should incorporate confidence building and to make students feel comfortable to take sexual histories from patients. The barrier caused by differences in culture or religion between a doctor and a patient may be overcome through cross cultural and cultural competency training. This is important for multi-faith, multi cultural societies such as Malaysia and other similar countries.

## Background

Sexual and reproductive health (SRH) is an important aspect of wellbeing. In 2002, the World Health Organization (WHO) defined sexual health as “a state of complete physical, mental and social wellbeing and not merely the absence of disease or infirmity in all matters relating to the reproductive system and to its functions and processes” [1]. This definition changed perceptions toward sexual health to be more holistic, incorporating various aspects of a person. In view of this definition, sexual health problems may present to doctors in various situations, such as sexually transmitted infection (STI), reproductive or pregnancy-related problems, HIV or AIDs, sexual violence, or sexual dysfunction.

STI is increasing worldwide. The WHO estimated the global incidence of STIs in 2008 in people aged 14–49 to be 498.9 million, compared with 300 million cases in 2006 [2, 3]. This is a global situation and includes upper-middle income countries [4] such as Malaysia. In a Malaysian national study conducted in 1994–1995, nearly 1.0% of 1,379 teenagers aged 13–19 years had engaged in sexual intercourse [5], and this prevalence had increased to 5.4% in 2001 [6] and 12.6% in 2005 [7]. “Baby dumping” is a national concern and a result of non-marital sexual relationships leading to unwanted pregnancies, which are culturally taboo in Malaysian society. Police statistics reported 517 cases of baby dumping from 2005 to February 2011 [8].

The true extent of STI in Malaysia is unknown. This is because of various factors, such as under-reporting, under-diagnosis, patients refusing to seek treatment, social embarrassment, and other factors [2]. The first case of HIV in Malaysia was reported in 1986, and, up to December 2006, there were 76,386 cases of HIV reported by the Ministry of Health, with 9,155 deaths [9, 10]. This has a social and economic impact upon the health service of the country.

For these reasons, it is important for doctors to be able to take a sexual history and explore patients’ sexual health problems to diagnose, manage, and counsel patients accordingly. It has been widely reported that doctors find it uncomfortable to approach the subject of sex and to probe into patients’ sexual practices [11, 12]. However, it has also been found that patients would like to discuss their sexual health problems with their doctors and prefer that their doctors initiate the subject [13]. Furthermore, patients would like to discuss these issues with doctors who have adequate knowledge and are comfortable addressing the patient’s sexual concerns [13]. Doctors who are comfortable in discussing sexual health issues were found to be more likely to uncover sexual health problems in patients [14, 15]. High awareness and positive attitudes among doctors are the starting point for the good delivery of sexual health care [16].

Undergraduate training has an important role in introducing sexual history taking to future doctors and in building their confidence and comfort level. Medical students often find it uncomfortable to take sexual histories and feel that they are inadequately trained [16]. In many countries, medical students still receive training in sexual history taking skills, assessment, and management that is variable, non-standardized, and inadequate [17]. Even in developed countries, such as in the UK, where the undergraduate training in sexual history taking has improved, it is still taught in specialist centers such as genitourinary clinics, and students find it difficult to raise the subject in general consultation [18, 19].

Medical undergraduate training in Malaysia was found to lack a standardized curriculum on sexual health and sexual history taking [20]. The purpose of this study was to assess the attitudes and perceptions of final-year medical students in Malaysia regarding sexual history taking and the training they receive from their medical schools.

## Methods

### Study design and population

A cross-sectional study was conducted at three medical schools in Malaysia: Universiti Teknologi MARA (UiTM), University Malaya (UM), and International Medical University (IMU). The study was conducted from May to November 2012 and used a newly developed questionnaire. Only final-year medical students were selected, because they have completed most of their training within the curriculum.

### Sampling method

All final-year medical undergraduate students were invited to participate in the study. Participation was on a voluntary basis, and confidentiality was assured. The students were briefed on the purpose of the study, and, for those who agreed to participate, written consent was obtained. Consent forms were collected separately, and the participants were then given the questionnaires to complete anonymously. A minimum sample size of 364 participants was calculated using a population survey sample size calculator, with a 95% confidence level and a 5% confidence interval. Ethical approval was obtained from the relevant institutional ethics committee of all three universities.

### Study tools: the questionnaire

We used a self-assessed questionnaire to measure the attitudes and perceptions of medical undergraduate students regarding sexual history taking and training. The questionnaire was designed for students to reflect upon and rate their perceptions regarding their own attitudes on sexual history taking as well as the training they received on the subject.

A review of published literature on SRH and sexual history taking was performed to identify domains to be included in the questionnaire. The attitude and skill domains were identified, and items were created to cover these areas. After the item pool was generated, a team of experts was formed to identify item redundancy, clarity, and content validity. The questionnaire was designed in the English language, as the medical curriculum is taught in English, and students were given the opportunity during the pilot study to give feedback and comment on their understanding of the questionnaire.

The finalized questionnaire consisted of (part 1) survey items and (part 2) demographic details. Part 1 was subdivided into three sections: section A comprised 16 questions to assess the students’ attitudes toward sexual history taking, section B contained eight items measuring the students’ perceptions of their own skills and training, and section C asked the students to rate the sexual history taking training they received from eight different specialties. In all three sections, respondents recorded responses on a five-point Likert scale, ranging from (1) strongly disagree to (5) strongly agree and including (3) neutral. The questionnaire was pilot tested with 38 final-year medical students prior to the main study. The Cronbach’s alpha of the questionnaire was 0.73, indicating that the questionnaire was reliable.

### Statistical analysis

Data analysis was conducted using SPSS, Version 20.0. The frequency distribution, measures of central tendencies, and measures of distribution were produced. A descriptive analysis was conducted for all sections. For the analysis, the Likert scale responses were grouped into three categories: “strongly agree” and “agree” were grouped as “agree”, “strongly disagree” and “disagree” were combined into “disagree”, and neutral remained. Using the mean score for each item, the Kruskal–Wallis nonparametric test was used to analyze the significance of variables with more than two response values (e.g., ethnicity), and the Mann–Whitney test was used to analyze the significance of variables with two response values (e.g., gender). The Mann–Whitney test uses z-scores below −1.96 and above 1.96 to reject the null hypothesis. Only significant items are reported in this study. The significant level was set at p = 0.05.

## Results

A total of 379 medical undergraduate final-year students participated in the study, with a response rate of 70% from the three medical schools. The response rate for men is 80% and the response rate for women is 65%. The demographic details of the participants are shown in Table 1.

**Table 1:** Demographic details of the participants

Variables	Frequency (%)	Mean (SD)
All subjects, *n* (%)	379 (100)	
Age		23.6 (0.65)
Gender^a^		
Male	144 (38.2)	
Female	233 (61.8)	
Race^a^		
Malay	214 (56.5)	
Chinese	120 (31.7)	
Indian	32 (8.4)	
Others	11 (2.9)	
Religious^a^		
Religious constituted	234 (61.2)	
Slightly religious	113 (29.8)	
Not religious	29 (7.7)	
Doctor in family^a^		
Yes	98 (26.0)	
No	278 (74.0)	
Sexual activity^a^		
Active	63 (16.8)	
No active	313 (82.6)	
Marital status^a^		
Single	356 (93.9)	
Married	21 (5.5)	

^a^Numbers not equal to *n* = 379 because of missing data.

Table 1 presents the demographic details of the participants. The sample comprised twice the number of females compared to male medical students. This increasing number of females within medical schools is reflected in other Malaysian studies [21, 22]. The ethnic distribution of the participants was representative of the Malaysian population, based on the 2009 population census [23].

Table 2 reports the participants’ attitudes toward sexual history taking. Participants agreed that they were interested in sexual health (76%) and realized the importance of doctors being able to take sexual histories (96%). Half of the participants felt unsure or uncomfortable discussing sexual health issues with patients. This is also reflected in the way that they responded to items gauging feelings of discomfort discussing sexual health issues with adolescents, patients of the other sex, and unmarried sexually active patients, as well as discussing sexual orientation. Cultural or religious differences between the doctor and the patient were identified as a potential barrier in discussing sexual health issues openly. Most participants were able to recognize their limitations in this subject and almost all of them respected patient confidentiality.

**Table 2:** Percentage and frequency of the participants’ responses on their attitudes toward sexual history taking

No		Disagree, frequency (%)	Neutral, frequency (%)	Agree, frequency (%)
1.	I am interested in learning about sexual health^a^	10 (1.0)	80 (21.0)	287 (76.0)
2.	I think that It is important for doctors to know how to take a sexual history	4 (1.0)	12 (3.0)	363 (96.0)
3.	I feel that a nurse can take better sexual history^a^	140 (36.7)	190 (50.0)	46 (12.3)
4.	I think that It is important to be nonjudgmental when taking a sexual history^a^	9 (2.0)	14 (4.0)	354 (94.0)
5.	I feel comfortable in discussing sexual health problems with patients^a^	64 (16.0)	127 (34.0)	186 (49.0)
6.	I feel comfortable discussing sexual health problems with adolescents^a^	69 (18.0)	122 (32.0)	187 (50.0)
7.	I feel comfortable discussing sexual health problems with patients of opposite gender^a^	110 (29.0)	124 (33.0)	143 (37.0)
8.	I feel comfortable discussing sexual health problems with unmarried but sexually active patients	65 (17.0)	110 (29.0)	204 (54.0)
9.	I feel comfortable in asking patients about their sexual orientation e.g. homosexuals^a^	102 (27.0)	122 (32.0)	153 (40.0)
10.	I feel comfortable in asking patients regarding their sexual practices e.g. “Are you sexually active?”, “Do you practice vaginal sex?”^a^	91 (24.0)	115 (30.0)	172 (45.0)
11.	I feel comfortable in taking a sexual history from patients who are uneasy in discussing sex^a^	175 (46.0)	129 (34.0)	72 (19.0)
12.	I feel that cultural differences are a barrier when discussing sexual health problems with patients^a^	76 (19.0)	107 (28.0)	195 (51.0)
13.	I feel that religious differences are a barrier when discussing sexual health problems with patients^a^	82 (22.0)	105 (28.0)	191 (51.0)
14.	I recognize my own limitations in discussing sexual health issues with patients	19 (4.8)	74 (20.0)	286 (75)
15.	I have thought about how my own attitudes, beliefs and values may affect my discussion of sexual health issues with patients^a^	27 (7.3)	93 (25.0)	258 (69.0)
16.	I believe that it is important to maintain patient confidentiality	6 (1.6)	14 (4.0)	359 (95.0)

^a^Numbers not equal to *n* = 379 because of missing data.

Table 3 reports participants’ perceptions of their skills and of the training that they receive on sexual history taking. Only 16.3% of participants identified sexual history taking as easy, and they were divided (30% disagreed and 27.4% agreed) on whether they perceived themselves as having adequate skills in performing the task.

**Table 3:** Percentage and frequency of participants’ perceptions of their skills and training received on sexual history taking

No		Disagree, frequency (%)	Neutral, frequency (%)	Agree, frequency (%)
1.	I find taking sexual history easy^a^	140 (37.0)	175 (46.0)	63 (16.3)
2.	I have adequate skills to take sexual history^a^	112 (30.0)	162 (43.0)	104 (27.4)
3.	I have adequate skills to put a patient at ease when discussing their sexual health issues^a^	110 (29.4)	152 (40.0)	116 (31.1)
4.	The training in my medical school prepares me to take a sexual history	88 (24.0)	121 (32.0)	170 (46.0)
5.	I have enough exposure as a medical student to take a sexual history from a real patient	111 (29.0)	127 (34.0)	141 (38.0)
6.	I have enough exposure as a medical student to take a sexual history from a simulated patient^a^	115 (30.0)	126 (33.0)	135 (35.0)
7.	I feel that there is not enough training in the medical school on how to discuss sexual health problems with patients^a^	83 (22.0)	120 (32.0)	175 (46.0)
8.	I feel that patients would like to discuss their sexual health problems with a doctor^a^	56 (15.0)	122 (32.0)	200 (53.0)

^a^Numbers not equal to *n* = 379 because of missing data.

With regard to the training received, only half felt that their medical school had prepared them for taking sexual histories. Less than half of the participants felt that had enough exposure as students to taking sexual histories from patients or simulated patients. Nearly half of the participants felt that they had not received adequate sexual history training in medical school.

In the analysis of factors associated with mean scores on attitude and skills items, only sex was found to be significant. Other factors, such as ethnicity, religion, having doctors in the family, sexual history, and marital status, were non-significant. Comparing findings by sex, males found taking sexual histories easier than did females (z = −3.293, p = 0.001). Males rated themselves as having adequate skills more frequently than did females (z = −3.326, p = 0.001), and, compared with females, males were better able to put patients at ease (z = −4.284, p = 0.0001).

Table 4 reports the participants’ perceptions of adequacy of training in sexual history taking received during their specialty postings. From the responses, obstetrics and gynecology attained the highest percentage scores (84%), followed by primary care medicine (77%) and general medicine (61%).

**Table 4:** Participants’ perceptions of adequacy of training on sexual history taking received during their specialty postings

Specialty	Agree frequency (%)	Neutral frequency (%)	Disagree frequency (%)	Mean score ± SD
Obstetrics and gynecology	217 (84)	44 (12)	18 (4.8)	3.10 ± 0.81
Primary care	291 (77)	72 (19)	16 (3.8)	2.94 ± 0.79
General medicine	232 (61)	102 (27)	44 (12.1)	2.60 ± 0.86
Psychiatry	185 (49)	117 (31)	73 (19)	2.35 ± 0.98
Public health	131 (35)	126 (33)	119 (32)	2.04 ± 1.02
General surgery	95 (25)	134 (35)	148 (39)	1.84 ± 0.94
Pediatrics	47 (12)	103 (27)	227 (60)	1.37 ± 0.98
Orthopedics	37 (9.5)	107 (28)	231 (61)	1.32 ± 0.88

## Discussion

This study has shown that medical students are interested and recognize the importance in attaining skills in sexual history taking. This is echoed in other studies conducted among medical students and practitioners [19, 24]. This is encouraging because it represents a positive attitude toward the subject and other studies have shown that high awareness and positive attitudes among doctors are the starting point for the good delivery of sexual health care [16].

This study has also highlighted students’ concerns, such as feeling uncomfortable discussing sexual health with patients and this is shared by medical students in other parts of the world [16, 25]. Consultations involving sexual health with adolescents, patients of the other sex, and unmarried sexually active patients, as well as discussions on sexual orientation are seen as barriers in taking sexual histories. These findings are similar to other studies where feeling of embarrassment, ill-preparedness, and the lack of a nonjudgmental approach may be barriers in sexual history taking [16, 20, 25–27]. It is clear that the training programs in medical schools need to address these issues, teaching students on how to handle uncomfortable situations and how to remain nonjudgmental.

Cultural and religious differences between the doctor and the patient were identified as a barrier in discussing sexual health. This finding is echoed in studies involving healthcare professionals [28, 29]. This is a significant finding within a multicultural society, where cultural and religious differences are inevitable. The concern is that, because of these barriers, doctors may shy away from taking sexual histories from patients and, thus, be unable to identify patients’ health needs. Cultural competency training has been shown to improve knowledge, attitudes, and skills of health professionals, as well as patient satisfaction [30], it is therefore recommended that such training could be incorporated within the sexual health undergraduate training module.

Nearly half of the student population in this study felt unprepared to take a sexual history and that the training they received was inadequate to provide them with the necessary skills. The importance of undergraduate training in taking sexual histories has been recognized in many medical schools around the world, but there are great differences in the extent to which this topic is covered [19, 20, 31]. In Malaysia, sexual history training remains an ad hoc topic covered by various specialties [20] such as obstetrics and gynecology, primary care medicine, and general medical postings. In this study, students regarded these specialties as better than others at preparing them for sexual history taking. This finding raises the question of whether these specialties should take the lead in training medical students on taking sexual histories or whether other specialties should also be encouraged to explore such training within their curriculum to give students a broader understanding of sexual health.

Similar to another study, student’s age, previous exposure to sex, marital status, and religiosity are not significantly associated with preparedness to take a sexual history [32]. Student’s sex did have a significant effect: men rated themselves as more adequately prepared than did women, possibly because of gender bias in self-rating questionnaires [33].

Various studies and methods have been introduced in other countries to improve undergraduate training in sexual history taking and sexual health. This includes student observation of history taking [34], student delivery of peer-led sex education [35], genitourinary attachments [19], the use of simulated patients, and workshops [20, 36]. These methods can be adopted as teaching and learning tools within a sexual history training program. The gaps identified from this study can be used to improve the delivery of sexual health training among medical schools in Malaysia which includes building confidence and to make students feel comfortable to take a sexual history from patients. Cross cultural training and cultural competency training can be incorporated within the curriculum to overcome the cultural and religious barriers between a doctor and a patient.

## Limitations

Our study has several limitations. The questionnaire was prepared in English rather than the local Malay language, and this may have affected the students’ perceptions of the questions and their responses. It is possible that not all respondents were completely fluent in English; Malay is the national language in Malaysia, whereas English is taught as a second language in schools. The researchers took this into account and conducted a pilot study as well as content validation on the questionnaire. The researchers also agreed that, because English is the language used for teaching and learning in medical schools in the country, students are expected to be able to understand and respond competently. Another possible limitation is that, although the students’ responses were self-reported and anonymous, bias may have resulted from students providing answers that they thought would be more socially and professionally acceptable. Students may also have wished to “please” the researchers with positive answers. Self-rating may introduce bias, although studies have found that students have the ability for self-assessment, and their accuracy increases at their later years of training [37].

## Conclusions

This study identified gaps in sexual health training among medical schools in Malaysia for undergraduate students. Lack of confidence in approaching the subject of sexual health, inadequate preparation were some of the gaps identified. The delivery of sexual health education program should incorporate confidence building and to make students feel comfortable to take a sexual history from patients. The barrier caused by differences in culture or religion between a doctor and a patient can be overcome through cross cultural and cultural competency training and this is important for multi-faith, multi cultural societies such as Malaysia and other similar countries.
